# L-Arginine Affects Aerobic Capacity and Muscle Metabolism in MELAS (Mitochondrial Encephalomyopathy, Lactic Acidosis and Stroke-Like Episodes) Syndrome

**DOI:** 10.1371/journal.pone.0127066

**Published:** 2015-05-20

**Authors:** Lance H. Rodan, Greg D. Wells, Laura Banks, Sara Thompson, Jane E. Schneiderman, Ingrid Tein

**Affiliations:** 1 Division of Neurology, Dept. of Pediatrics, Hospital for Sick Children, University of Toronto, Toronto, Ont., Canada, M5G 1X8; 2 Physiology and Experimental Medicine Program, Hospital for Sick Children, University of Toronto, Toronto, Ont., Canada, M5G 1X8; 3 Faculty of Kinesiology and Physical Education, University of Toronto, Toronto, Ont., Canada, M5G 1X8; 4 Dept. of Laboratory Medicine and Pathobiology, University of Toronto, Toronto, Ont., Canada, M5G 1X8; University of Sevilla, SPAIN

## Abstract

**Objective:**

To study the effects of L-arginine (L-Arg) on total body aerobic capacity and muscle metabolism as assessed by ^31^Phosphorus Magnetic Resonance Spectroscopy (^31^P-MRS) in patients with MELAS (Mitochondrial Encephalomyopathy with Lactic Acidosis and Stroke-like episodes) syndrome.

**Methods:**

We performed a case control study in 3 MELAS siblings (m.3243A>G tRNA^leu(UUR)^ in MTTL1 gene) with different % blood mutant mtDNA to evaluate total body maximal aerobic capacity (VO_2peak_) using graded cycle ergometry and muscle metabolism using ^31^P-MRS. We then ran a clinical trial pilot study in MELAS sibs to assess response of these parameters to single dose and a 6-week steady-state trial of oral L-Arginine.

**Results:**

At baseline (no L-Arg), MELAS had lower serum Arg (p = 0.001). On ^31^P-MRS muscle at rest, MELAS subjects had increased phosphocreatine (PCr) (p = 0.05), decreased ATP (p = 0.018), and decreased intracellular Mg^2+^ (p = 0.0002) when compared to matched controls. With L-arginine therapy, the following trends were noted in MELAS siblings on cycle ergometry: (1) increase in mean % maximum work at anaerobic threshold (AT) (2) increase in % maximum heart rate at AT (3) small increase in VO_2peak_. On *^31^P-MRS *the following mean trends were noted: (1) A blunted decrease in pH after exercise (less acidosis) (2) increase in Pi/PCr ratio (ADP) suggesting increased work capacity (3) a faster half time of PCr recovery (marker of mitochondrial activity) following 5 minutes of moderate intensity exercise (4) increase in torque.

**Significance:**

These results suggest an improvement in aerobic capacity and muscle metabolism in MELAS subjects in response to supplementation with L-Arg. Intramyocellular hypomagnesemia is a novel finding that warrants further study.

**Classification of Evidence:**

Class III evidence that L-arginine improves aerobic capacity and muscle metabolism in MELAS subjects.

**Trial Registration:**

ClinicalTrials.gov NCT01603446.

## Introduction

Mitochondrial encephalomyopathy, lactic acidosis, and stroke-like episodes (MELAS) syndrome is one of the most common and devastating mitochondrial diseases. MELAS syndrome is associated with a myriad of neurological and systemic symptoms, including myopathy, exercise intolerance and stroke-like episodes [1]. Unique to MELAS syndrome, and presumed to underlie the stroke-like episodes, is a functional vasculopathy resulting from abnormal mitochondria in vascular smooth muscle and endothelial cells [2]. It is uncertain whether this vasculopathy plays a role in the myopathy and exercise intolerance these patients usually manifest.

Recent work has demonstrated a beneficial effect of L-arginine therapy in MELAS for treatment and prevention of stroke-like episodes [3]. The same authors have also shown increased perfusion of skeletal muscles on brachial artery Doppler studies following supplementation with L-arginine [4]. The mechanism (s) by which arginine effects its benefit have not been fully elucidated, although it is proposed to increase nitric oxide (NO) mediated vasodilation.

A number of techniques have been utilized to assess metabolic function of muscle in patients with mitochondrial and other metabolic myopathies. ^31^Phosphorus Magnetic Resonance Spectroscopy (^31^P-MRS) of muscle measures levels of phosphocreatine, inorganic phosphate, ATP, and pH non-invasively and can be used to assess the function of creatine kinase, anaerobic glycolysis, and oxidative phosphorylation bioenergetic pathways [5]. Subjects with mitochondrial myopathy have lower baseline levels of creatine phosphate, higher inorganic phosphate, and lower pH. Following exercise, they show a delay in their recovery of phosphocreatine stores [6].

Another technique employed in the analysis of mitochondrial myopathy is maximal incremental exercise testing on a cycle ergometer. Compared to healthy controls, patients with mitochondrial myopathy cross their anaerobic threshold after less time and at lower work levels [7] and are limited in their ability to extract available oxygen from blood [8].

The therapeutic efficacy of L-arginine in aerobic capacity and muscle metabolism is yet to be determined. The purpose of this study was to evaluate the effects of L-arginine supplementation *in vivo* on total body aerobic capacity and on muscle metabolism in a family of siblings, with different percentage heteroplasmy of the m.3243A>G tRNA^Leu(UUR)^ MELAS mutation in MTTL1 gene in blood, using maximal graded cycle ergometry and ^31^P-MRS of muscle respectively. We anticipate that even though there will be a decrease in oxygen uptake by exercising muscle due to defective oxidative phosphorylation, there may be an additional defect related to impaired peripheral vasodilatation, due to endothelial dysfunction of small muscle arterioles, which may respond to L-arginine. Alternatively, there may be a primary metabolic benefit to L-arginine supplementation on muscle mitochondrial metabolism, such as improved cellular anaplerosis. Detailed study of this family provides us with the opportunity to compare their clinical features with their exercise physiology, and to gain insight into the therapeutic targets and relative response thresholds to L-arginine therapy in this unique cohort which shares a common genetic background.

## Materials and Methods

### Study Methodology

We employed a case control methodology for the comparison of exercise parameters in MELAS subjects and matched controls to evaluate aerobic capacity and muscle metabolism *in vivo* using maximal incremental graded cycle ergometry and ^31^P-MRS of muscle respectively. We subsequently used a non-randomized clinical trial pilot study design to assess the response to L-arginine in MELAS subjects only. The protocol for this trial and supporting CONSORT checklist are available as supporting information; see S1 TREND Checklist and S1 Protocol. This non-randomized pilot efficacy study was registered on the ClinicalTrials.gov (NIH) website under identifier: NCT01603446. Ethics approval was obtained from the Research Ethics Board of the Hospital for Sick Children, Toronto, Canada. A data safety monitoring committee was set in place. Written informed consent was obtained from all participants and from the next of kin on behalf of all minors enrolled in the study using formal consent forms approved by the Research Ethics Board of the Hospital for Sick Children. All clinical investigations were conducted according to the principles expressed in the Declaration of Helsinki.

### Subjects

Three siblings (two female) aged 17, 21 and 22 years with genetically confirmed MELAS syndrome associated with the m.3243A>G tRNA^Leu(UUR)^ mutation were recruited from the Neurometabolic clinic at the Hospital for Sick Children, Toronto, Canada. Four healthy age and sex-matched controls (three females, one male) living in Toronto were recruited through posted advertisements, approved by the Research Ethics Board, at the Hospital for Sick Children, Toronto and the University of Toronto by referral and by self-selection. Female patients were also matched to controls for timing of menstrual cycle, and estradiol levels were measured, as estrogen appears to alter myogenic tone by increasing cerebrovascular NO production and/or action [9]. Healthy controls had no ongoing medical conditions that could affect exercise performance (neuromuscular or other neurological disease, genetic metabolic disorder, cardiac or pulmonary disease, hypertension, or anemia) and were taking no medications. Healthy controls were also screened prior to study entry for a normal baseline physical examination and blood pressure measurement. The studies were performed and the data was collected at the Hospital for Sick Children in Toronto. The active study period including recruitment of subjects, clinical testing and follow up was completed in 14 months between March 2012 and May 2013.

### Study design

**Fig 1:**
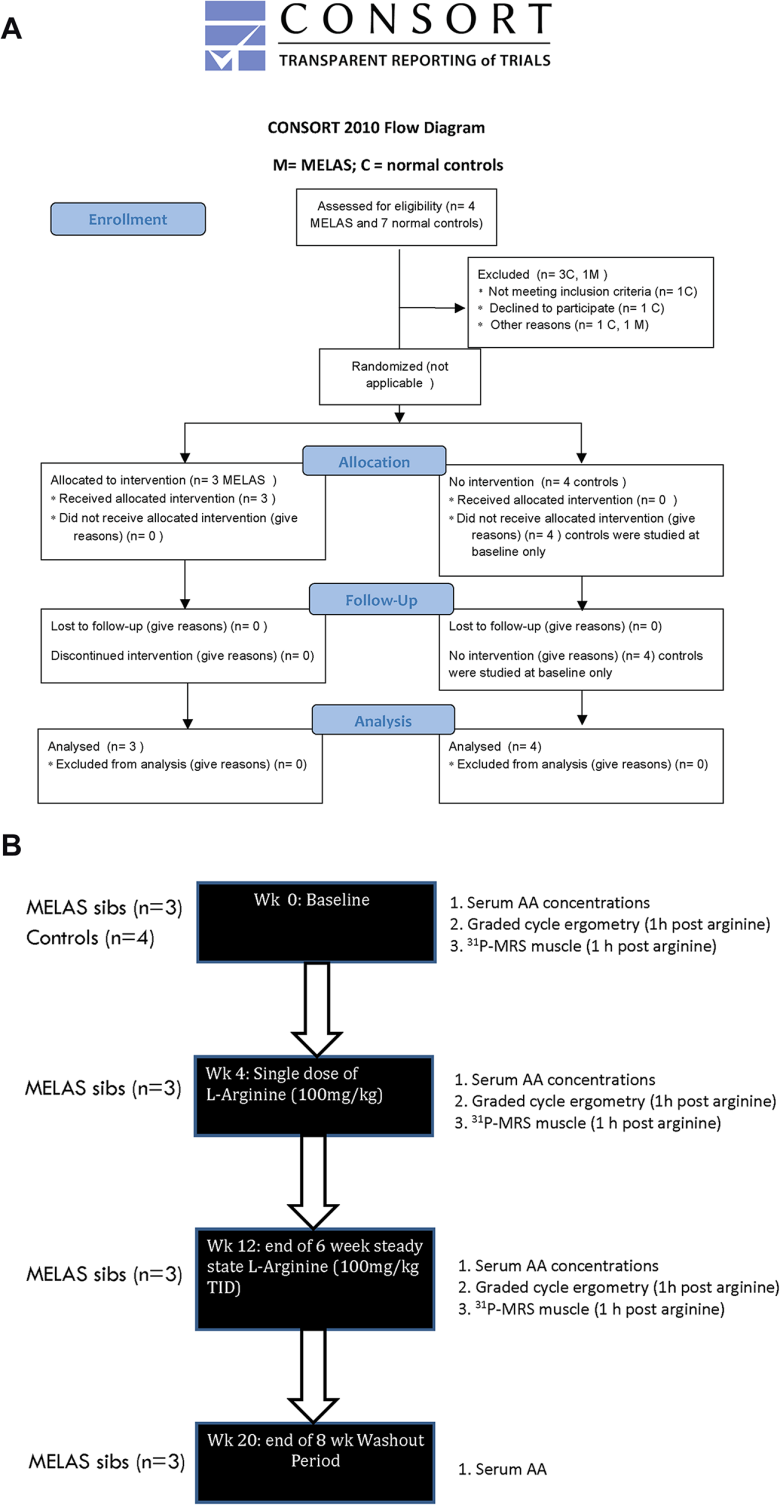
Flow Diagrams for the MELAS/L-arginine study (A) Consort 2010 Flow Diagram (B) Schema for MELAS/L-arginine Study Protocol.

A consort flow diagram is given in **Fig 1A** and a study flow diagram is represented in **Fig 1B**. MELAS subjects had normal cardiac function. At baseline, both MELAS subjects and controls underwent a complete neurological examination, pulmonary function tests, a Habitual Activity Estimation Scale (HAES), and laboratory studies including complete blood count, electrolytes, renal functions, liver functions, serum glucose, PT, INR, carnitine total and free, CK, lactate, and serum quantitative amino acids and urine organic acids. Serum amino acids were measured at four time-points over the day, both pre- and post-prandially, to better assess average amino acid levels. The control females and the control male underwent only the baseline studies, completed in one day, which were conducted on separate days from the MELAS subjects. The MELAS subjects underwent the studies in tandem on the same day. Studies were conducted at 4 week intervals to coincide with the same time point in menstrual cycle. Exercise studies in MELAS subjects and controls included maximal graded cycle ergometry and ^31^P MRS measurements in quadriceps muscle (before and after exercise). On week 4, MELAS patients only were given a single dose of oral L-arginine (100 mg/kg) mixed in solution by the clinical research trial nurse in the Physiology Research Unit at the Hospital for Sick Children, Toronto. Serum amino acids were measured following administration (4 time points: 1 hour post and 3–5 hours post). Maximal graded cycle ergometry and ^31^P-MRS measurements of quadriceps muscle were repeated one hour post L-arginine to ensure peak serum arginine concentrations at the time of exercise. On week 6, MELAS patients were commenced on a 6 week, steady state trial of oral L-arginine at 100mg/kg three times daily as per Koga et al [4]. At week 12 (MELAS patients only) serum amino acids were again repeated (4 time points) along with baseline bloodwork. Maximal graded cycle ergometry and ^31^P MRS measurements of quadriceps muscle were again repeated one hour post- oral L-arginine administration. Finally, at week 20 (MELAS patients only), following an 8 week wash-out period, serum amino acids were repeated along with the baseline bloodwork. High grade, highly purified NOW foods commercial Natural Health Product L-Arginine Powder, NPN 80002672, Bloomingdale, Illinois, which was approved for use on the Canadian market by Health Canada, Natural Health Products Directorate, was used. All subjects received a small incentive for their participation.

### Parameters of assessment

Height and weight (model 555; SR Instruments, Tonawanda, NY) were measured, and lean body mass was calculated based on body composition from MRI analysis [10]. Pulmonary function (VMax20 Pulmonary Spirometry Instrument; SensorMedics, Yorba Linda, CA) was determined according to standard spirometric techniques [11], and expressed as a percentage of predicted value for height and gender [12]. Subjects performed an incremental cycling test to determine maximal aerobic capacity [13] and completed the Habitual Activity Estimation Scale [14] as a validated estimation of activity levels in children with chronic disease [15]. Maximal oxygen uptake (VO2max) was expressed as a percentage of predicted norms for VO2max [16].

### Magnetic resonance imaging and spectroscopy tests

All magnetic resonance images (MRI) and ^31^P-MRS data were collected on a Siemens Magnetom 3T Tim Trio Vb17 at the Hospital for Sick Children [17]. ^31^P-MRS data were collected at rest and post exercise. Participants lay supine in the MR apparatus and performed exercises on a calibrated nonmagnetic up-down ergometer (Lode AEI Technologies). The lower extremities of the subjects were at the center of the magnet bore of the MRI scanner. By convention, the non-dominant leg was used for testing. Motion due to movement of the muscle in relation to the coil was minimized by securing the coil in a fixed position midway between the hip and the knee with Velcro straps. The ergometer automatically controlled power output by adjusting resistance in relationship to the subjects’ freely chosen movement frequency. In this way, exercise was controlled for power output, as relative work rate is an important factor in the determination of the metabolic pathways used for ATP generation during exercise and recovery. Watts and repetitions per minute (rpm) of the ergometer were recorded every 5 s during exercise.

Representative measurements were taken from the vastus lateralis muscle (although the ergometer movement required the use of vastus medialis, lateralis, intermedius, and rectus femoris muscles). The data collection involved MRI followed by shimming (calibration) and ^31^P-MRS spectroscopy data acquisition.

**Fig 2:**
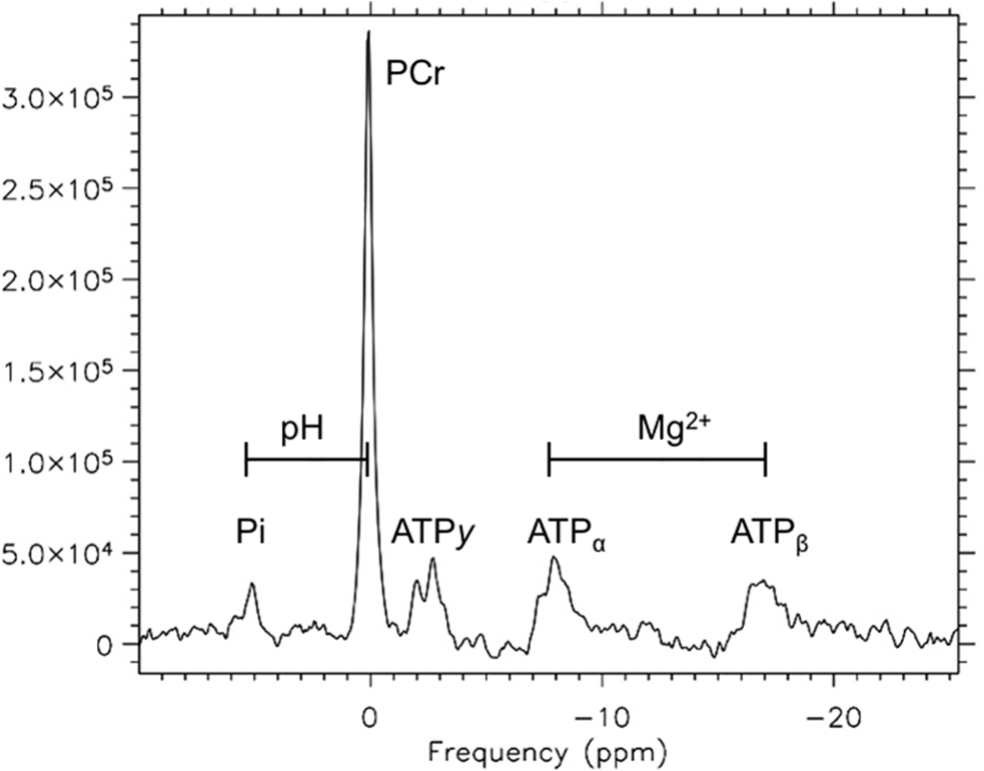
A typical spectrum acquired using ^31^P-MRS at rest from a healthy control subject. The peaks are representative of the concentrations of Pi, PCr and ATP. The pH and concentration of [Mg^**2+**^] can be calculated from the chemical shift between metabolites as indicated. Adapted from Pediatr Res 69 (1); pp 41, Fig 2 (2011) under a CC BY license, with permission from the Nature Publishing Group. [18]

**Fig 3:**
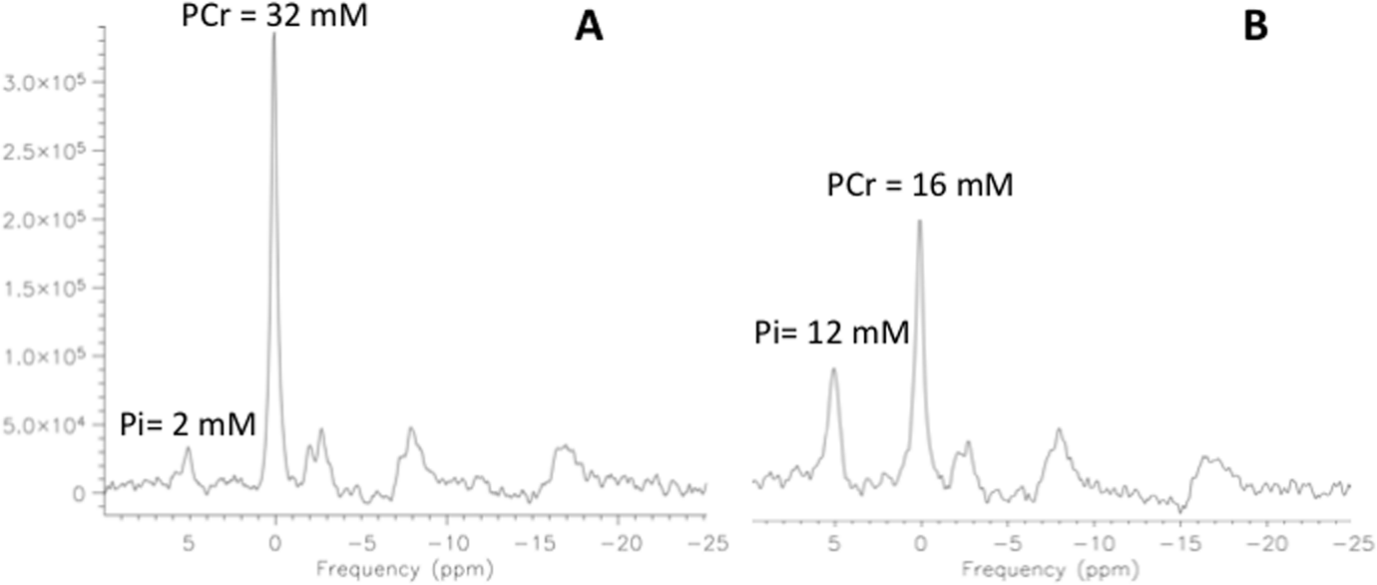
Typical spectra acquired using ^31^P-MRS before (A) and following exercise (B) from a healthy control subject. Note the changes in the Pi and PCr peaks. Reprinted from Pediatr Res 69 (1); pp 42, Fig 3 (2011) under a CC BY license, with permission from the Nature Publishing Group. [18]

For spectroscopy measurements, sequential ^31^P-MRS spectra were obtained under partially saturated conditions with the following parameters: FID-acquire sequence with two step phase cycling, 40° flip angle, TR 1000 ms, 1024 vector size, pulse duration 0.25 ms and 8 averages, total acquisition time = 8 s per spectrum. Spectral analyses were performed using commercial software (Syngo Siemens Germany). **Fig 2** [18] shows a typical spectrum acquired at rest, and **Fig 3** [18] shows pre- (**A**) and post- (**B**) exercise changes. Both figures are single spectra derived from four pulses as described in the methods mentioned above. Resting data presented in this article are based on the average metabolite values determined from eight resting scans.

A curve using nonlinear least squares analysis, based on Gaussian line shapes, was used to calculate the areas under the Pi, PCr, and ß-ATP peaks [19]. ^31^P metabolite concentrations were calculated by normalizing total muscle phosphate to 41.3 mmol/L [20]. Intracellular pH was calculated for each spectrum based on the chemical shift difference between PCr and Pi [21]. The cytosolic [Mg2+] was calculated from the chemical shift of ß-ATP measured from the resonance of PCr, and this information was used to correct calculated pH for changes in [Mg2+] [22]. The time constant of the recovery rate of PCr was calculated during recovery after each exercise bout using an exponential curve fit and was considered to be an index of aerobic mitochondrial metabolism.

### MRS exercise protocols

Three different exercise protocols were used to probe bioenergetic function during exercise bouts that are typical of different intensity activities. Participants performed a 30-s bout of maximal exercise to evaluate the physiological response to short bursts of intense activity representing the utilization of readily available muscle high energy phosphagens [23]. Average wattage produced was recorded and used to establish the intensity of subsequent exercise. The subjects recovered for 5 min before performing a 60-s exercise bout to evaluate the response to sustained intense exercise at a work rate equivalent to 85% of the mean watts achieved during the 30-s bout, representing primarily anaerobic exercise. After recovery, participants performed 10 bouts of 30 s exercise, separated by 15 s of rest, at 65% of the work rate during the 30-s exercise bout to evaluate the metabolic response to repeated bouts of moderate intensity activity representing primarily aerobic exercise. Adequate recovery after exercise was ensured by monitoring the phosphocreatine (PCr) and inorganic phosphate (Pi) peaks to ensure that they had returned to resting levels before beginning the subsequent exercise trial.

### Statistical Analysis

Statistical analysis comparing MELAS subjects to controls was conducted using an unpaired student’s t-test (normal distribution of samples) as well as nonparametric analysis (non-normal distribution) using either the Wilcoxon-Mann-Whitney test or Kruskal-Wallis test. A paired t-test was used to compare parameters for MELAS subjects before and after arginine administration. A one-way repeated measures ANOVA analysis (factor: group) was used to assess the 5-min exercise test results as 10 samples per patient were collected during this test. Statistical significance was set at p < 0.05.

## Results

### Patient characteristics

ELAS and control subject characteristics are summarized in **Table 1**. Four MELAS siblings were screened for the study and three were found to be eligible. Seven control subjects were screened for the study and four were found to be eligible. All study subjects completed the study. MELAS subject 3 had the highest percentage of mutant mtDNA in blood and was the only one with a history of prior stroke-like episodes. MELAS subject 1 had the lowest percentage of mutant mtDNA in blood and was asymptomatic by self-report. Mean habitual physical activity level based on the HAES questionnaire was greater in the control group; furthermore, controls 2 and 3 were engaged in regular recreational aerobic sports activities. All MELAS subjects and controls had normal peak power (MRC grade 5/5) on specific muscle group testing in all major muscles. There was no clinical evidence of peripheral neuropathy in any of the subjects. Serum creatine phosphokinase (CPK), hemoglobin, electrolytes, and serum carnitine concentrations were normal in all subjects and similar between groups. MELAS subjects had significantly lower serum arginine concentrations (53 ± 11 μmol/L) than study controls (94 ± 18 μmol/L; p = 0.001), although levels remained in the normal range. There were no significant differences between MELAS subjects and controls with respect to serum ornithine and citrulline concentrations. L-arginine supplementation successfully increased serum arginine concentrations in MELAS subjects (76 to 230 μmol/L). There were no adverse events related to the study.

**Table 1:** Clinical and neuroimaging features in MELAS cohort and healthy study controls.

Subject	Age	Gender	Ht (cm)	Wt (kg)	FVC (% predicted) at baseline	FEV1 (% predicted) at baseline	Oxygen saturation % in room air (normal > 95%)	% mutant mtDNA (blood)	Clinical and neuroimaging deficits	MOCA score
MELAS 1	22	F	164.1	48.0	98	102	normal	35	None	30/30
MELAS 2	21	F	156.8	44.6	74	87	normal	41	Sensorineural hearing loss, Minimal cerebral atrophy	26/30
MELAS 3	17	M	168.0	42.6	N/A	N/A	normal	59	Sensorineural hearing loss, Right hemianopsia, Left > right parieto/occipital SLEs	10/30
Control 1	22	F	161.0	62.0	86	100	normal	-	None	25/30
Control 2	21	F	163.0	61.5	104	103	normal	-	None	30/30
Control 3	21	F	172.0	58.2	70	83	normal	-	None	28/30
Control 4	17	M	181.0	89.4	96	88	normal	-	Retrocerebellar arachnoid cyst	28/30

**Key**: FVC = forced expiratory vital capacity is the volume change of the lung between a full inspiration to total lung capacity and a maximal expiration to residual volume; FEV1 = forced expiratory volume is the volume exhaled during the first second of a forced expiratory maneuver started from the level of total lung capacity; MOCA = Montreal Cognitive Assessment which is a cognitive screening test—a score of ≥ 26 is considered normal; N/A = not done due to difficulty with cooperation; SLEs = stroke-like episodes.

### Baseline graded cycle ergometry in MELAS subjects and controls

Mean VO2_peak_ and percentage of VO2_peak_ predicted reached during exercise was lower in MELAS subjects compared to controls (1.04 +/- 0.32 L/min compared to 2.31 +/- 0.71 L/min (p = 0.036) and 50 +/- 10.5% compared to 87 +/- 21.2% (p = 0.042 using an unpaired T-test for normal population distribution) respectively). This statistical significance was also confirmed using the Wilcoxon-Mann-Whitney test which is a nonparametric test used for non-normal populations. Additional parameters summarized in **Table 2** did not reach statistical significance with the exception of the VE/VCO2 which was higher in the MELAS subjects compared to controls (33.0 ± 5.2 compared to 26.7 ± 2.1; p = 0.051) using the Wilcoxon-Mann-Whitney test but not the t-test (p = 0.069).

**Table 2:** Total body aerobic capacity as measured by maximal graded cycle ergometry in healthy controls and MELAS subjects and effect of single dose and steady-state L-arginine therapy in MELAS subjects.

Subject	Time	% maximum work at AT	Peak work (Watts/kg)	% maximum heart rate at AT	VO2 peak L/min	VO2max ml/kg/min	VO2 max (% pred) ^@^	% VO2 max at AT	VE/VO2	VE/VCO2	RQ peak
MELAS 1	1	33 (30/90)	1.87 (90/48)	68 (121/178)	1.21	25.2	61	47	30	30	1.30
	2	44 (40/90)	1.90 (90/47.3)	72 (130/180)	1.07	22.6	54	56	32	31	1.39
	3	44 (40/90)	1.87 (90/47.9)	76 (138/181)	1.26	26.3	64	43	33	32	1.37
MELAS 2	1	29 (20/68)	1.52 (68/44.6)	70 (124/176)	0.68	15.3	40	61	43	39	1.31
	2	57 (40/70)	1.56 (70/44.8)	84 (143/171)	0.84	18.8	49	55	36	34	1.54
	3	28 (20/70)	1.51 (70/46.3)	73 (117/160)	0.66	14.3	38	48	37	33	1.60
MELAS 3	1	60 (60/100)	2.34 (100/42.6)	92 (155/169)	1.25	29.4	49	66	32	30	1.27
	2	62 (50/80)	1.88 (80/42.4)	83 (136/164)	1.18	27.8	44	95	30	30	1.19
	3	71 (70/98)	2.32 (98/42.1)	97 (160/165)	1.32	31.4	50	83	37	32	1.26
Baseline MELAS mean ± SD	1	40.7 ± 16.8	1.91 ± 0.41	76.7 ± 13.3	1.04 ± 0.32	23.3 ± 7.2	50.0 ± 10.5	58.0 ± 9.8	35 ± 7.0	33.0 ± 5.2	1.29 ± 0.02
Single dose MELAS mean ± SD	2	54.3 ± 9.3	1.78 ± 0.19	79.7 ± 6.6	1.03 ± 0.17	23.1 ± 4.5	49.0 ± 5.0	68.7 ± 22.8	32.7 ± 3.06	31.7 ± 2.08	1.37 ± 0.17
T-test * P value		0.21	0.51	0.70	0.87	0.91	0.86	0.40	0.46	0.55	0.46
Steady state MELAS mean ± SD	3	47.7 ± 21.7	1.90 ± 0.40	82.0 ± 13.0	1.08 ± 0.36	24.0 ± 8.8	50.7 ± 13.0	58.0 ± 21.8	35.7 ± 2.31	32.33 ± 0.58	1.41 ± 0.17
T-test * P value		0.22	0.98	0.067	0.34	0.51	0.69	1.00	0.86	0.82	0.32
Controls											
C1	1	57 (60/105)	1.69 (105/62)	88 (172/196)	1.33	21.4	62	86	30	29	1.2
C2	1	83 (150/180)	2.92 (180/61.5)	84 (152/180)	2.32	37.7	104	83	25	25	1.3
C3	1	71 (150/210)	3.60 (210/58.2)	92 (175/190)	2.59	44.5	105	70	27	27	1.15
C4	1	50 (105/210)	2.35 (210/89.4)	68 (136/201)	2.99	33.4	76	60	27	26	1.16
Control Mean ± SD		65.2 ± 14.7	2.64 ± 0.81	83 ± 10.5	2.31 ± 0.71	34.2 ± 9.7	86.7 ± 21.2	75.7 ± 12.0	27 ± 2.1	26.7 ± 1.7	1.20 ± 0.07
T-test ** P value		0.094	0.220	0.518	0.036 ***	0.164	0.042 ***	0.107	0.083	0.0689	0.0816
Wilcoxon-Mann-Whitney Test ^#^		0.216	0.216	0.860	0.051***	0.216	0.051***	0.216	0.077	0.051***	0.157

**Key: Work—**This is a physical quantification of the force operating on a mass that causes it to change its location.

**% maximum work at AT** (anaerobic threshold) is the work in watts at the AT/maximum work in watts achieved

**Peak work** is the maximum work in watts achieved/ weight in kg; **% maximum heart rate at AT** (anaerobic threshold) is the heart rate at the anaerobic threshold/maximum heart rate achieved; **VO2**—Amount of Oxygen taken up at the lungs, usually expressed in ml/kg/min. To all intents and purposes this is equal to the uptake at the cellular level (QO2) as there is minimal facility in the body to store oxygen. **VCO2**—Amount of Carbon dioxide exhaled, usually expressed in ml/kg/min. This is not the same as cellular CO2 production (QCO2) except in steady state. A steady state is not achieved during exercise testing with a ramp protocol. Hyperventilation increases VCO2 but not QCO2. **VO2 Max**- The maximum amount of oxygen the body can use expressed as ml/kg/min. It is defined as the point where the VO2 plateaus despite an increase in work rate. It is not the same as Peak VO2. The value is different for different forms of ergometry. The true VO2 max has no volitional component as it represents a physiological endpoint. **VE/VO2** = ventilatory equivalent for oxygen and is the ratio of minute ventilation to oxygen uptake by the lungs; **VE/VCO2** = ventilatory equivalent for carbon dioxide and is the ratio of minute ventilation to carbon dioxide excretion by the lungs; **Anaerobic threshold (AT)** is the timepoint at which the VE/VCO2 and VE/VO2 cross which occurs ~ at a respiratory quotient (RQ) of 1.0; Anaerobic Threshold, is usually expressed in mls. of oxygen uptake per kg body weight per minute. This is the oxygen uptake (VO2) at which anaerobic ATP synthesis supplements (not replaces) aerobic ATP synthesis and may reflect oxygen demand exceeding supply with subsequent production of lactic acid. It is thus an objective marker of exercise capacity/physiological reserve. In normal subjects it occurs at 35–80% of VO2 max.

**VO2/HR** = O2 pulse (O2 consumption per heart beat)

1 = baseline; 2 = single dose L-arginine; 3 = steady state L-arginine X 6 wks; SD = standard deviation

@ Maximal oxygen uptake (VO2max) was expressed as a percentage of predicted norms for VO2max [16]

* comparison of MELAS baseline versus MELAS single dose or MELAS baseline versus MELAS steady-state L-arginine therapy using paired T-test with two-tailed P value

** comparison of baseline MELAS versus control group using Unpaired T-test with two-tailed P value

*** statistically significant

^#^ comparison of baseline MELAS versus control group using the Wilcoxon-Mann-Whitney test or Wilcoxon rank-sum test which is a nonparametric test of the null hypothesis that two populations are the same against an alternative hypothesis which is used for non-normal population distributions

### Graded cycle ergometry in MELAS subjects following single dose and steady state arginine supplementation

Single dose and maintenance dosing of L-arginine appeared to normalize a number of exercise parameters on maximal graded cycle ergometry compared to baseline, although none of these reached statistical significance. Following single dose L-arginine, MELAS subjects crossed the anaerobic threshold at a higher percentage of maximum work (mean 54.3 compared to 40.7, a relative increase of 133%; p = 0.21) and heart rate (mean 79.7 compared to 76.7; p = 0.70). Also, the percentage of VO2peak reached at anaerobic threshold was increased (mean 68.7 compared to 58, a relative increase of 118%; p = 0.40), as was peak respiratory quotient (mean 1.37 compared to 1.29, a relative increase of 106%; p = 0.46). Maintenance dosing produced similar changes to single dose L-arginine, with the exception of the mean percentage of VO2peak at the anaerobic threshold, which was similar to baseline values. With maintenance dosing, there was also a higher percentance of maximum heart rate at the anaerobic threshold (mean 82.0 compared to 76.7, a relative increase of 107%; p = 0.067), nearing statistical significance.

When the results from single and maintenance dosing of arginine were averaged, the percentage of maximum work at the anaerobic threshold was significantly increased from baseline (51% compared to 40.7%, a relative increase of 125%; p = 0.037).

### Baseline ^31^P MRS in MELAS subjects and controls.

Parameters for MELAS subjects and controls at rest are summarized in **Table 3**. Compared to healthy controls, phosphocreatine (PCr) levels were elevated in MELAS subjects (p = 0.055), ATP levels were decreased (p = 0.018), and PCr/ATP ratio was elevated (p = 0.017) using an unpaired t-test. This statistical significance was not confirmed on nonparametric analysis using the Kruskal-Wallis test. The concentration of intracellular magnesium [Mg2+] in MELAS subjects was almost half that of controls (p = 0.0002), and the corresponding concentration of free or paracellular Mg (pMg) in MELAS subjects was almost double that of controls (p = 0.0001) and this statistical significance was also confirmed on nonparametric analysis.

**Table 3:** Baseline muscle metabolism (no L-arginine) during rest and different exercise regimens in MELAS subjects versus healthy control subjects as measured by ^31^P-MRS (mean ± SD).

Rest	MELAS patients	Controls	P (t-test)	Kruskal-Wallis Test
PCr	**30.6 ± 1.8**	**27.0 ± 2.0**	**0.055** *	0.284
Pi	2.44 ± 0.40	2.69 ± 0.49	0.508	0.723
Sum ATP	**6.52 ± 0.81**	**9.38 ± 1.22**	**0.018** *	0.153
PCr/ATP	**4.75 ± 0.79**	**2.93 ± 0.59**	**0.017** *	
pH	6.87 ± 0.06	6.86 ± 0.005	0.888	0.056
pMg	**2.87 ± 0.10**	**1.50 ± 0.16**	**0.0001** *	**0.032** *
[Mg^2+^]	**1.45 ± 0.28**	**2.83 ± 0.06**	**0.0002** *	**0.032** *
Pi/PCr (ADP)	0.08 ± 0.02	0.10 ± 0.02	0.178	0.475
**30 s**				
PCrRec ½ (s)	38.3 ± 12.0	24.0 ± 2.79	0.063	0.153
Pi/PCr (ADP)	0.31 ± 0.20	0.41 ± 0.16	0.52	0.463
HEP power	0.23 ± 0.18	0.26 ± 0.10	0.78	0.589
HEP dpH	0.16 ± 0.11	0.25 ± 0.04	0.18	**0.031** *
Watts/RPM	0/11.5	15.7/15		
**60s**				
AnPwr	0.14 ± 0.03	0.11 ± 0.04	0.39	0.207
dpH	0.55 ± 0.33	0.74 ± 0.27	0.42	0.281
Pi (B)	7.78 ± 2.61	8.37 ± 1.85	0.74	0.721
Pi/PCr (ADP)	0.47 ± 0.27	0.53 ± 0.23	0.76	0.723
PCrRec ½ (s)	29.3 ± 11.0	27.0 ± 0.76	0.69	0.475
Watts/RPM	8.2/13	12.2/15.5		
**5 min**				
ATP(Prod)CK	0.16 ± 0.06	0.09 ± 0.02	0.092	**0.032** *
ATP(Prod)Ox	0.31 ± 0.11	0.19 ± 0.03	0.086	**0.032** *
ATP(Prod)An	0.75 ± 0.42	0.42 ± 0.05	0.18	**0.032** *
ATP(Prod)tot	1.22 ± 0.60	0.70 ± 0.02	0.13	**0.032** *
dpH	0.32 ± 0.21	0.18 ± 0.08	0.29	0.475
Pi/PCr (ADP)	0.95 ± 0.89	0.45 ± 0.02	0.28	**0.032** *
PCrRec ½ (s)	64.4 ± 35.0	35.3 ± 8.83	0.16	**0.032** *
Watts/RPM	4.2/11.2	9.75/13.5		

Key:

AnPwr = anaerobic power; ATP = adenosine triphosphate

ATPAn = ATP anaerobic; ATPOx = ATP oxidative; ATPtot = ATP total

HEP = high energy phosphates; [Mg2+] = intracellular magnesium

pMg = free or paracellular magnesium and is calculated by the formula—log_10_[Mg2+]

Pi = inorganic phosphate; Pi/PCr ratio = ADP ratio

PCr = phosphocreatine; PCrRec 1/2 = halftime of phosphocreatine recovery in seconds

RPM = rotations per minute; SD = standard deviation

P is two-tailed P-value of unpaired t-test

* statistically significant result

Kruskal-Wallis one-way analysis of variance is a non-parametric method for testing whether samples originate from the same distribution and is used for comparing two or more samples that are independent and that may have different sample sizes (Pr > Chi-square)

^31^P-MRS parameters for MELAS subjects and controls after 30 seconds, 60 seconds, and 5 minutes of exercise are summarized in **Table 3**. Controls were able to exercise with significantly greater torque than MELAS subjects. None of the other parameters reached statistical significance, including phosphocreatine recovery using an unpaired t-test. However, on nonparametric Kruskal Wallis analysis, there was a significant delay in phosphocreatine recovery (p = 0.032) and unexplained increase in total ATP production (p = 0.032) in the MELAS subjects compared to the healthy controls.

### ^31^P MRS in MELAS subjects following single dose and steady state arginine supplementation (Table 4)

#### Single dose L-arginine

At rest there was a significant increase in Pi/PCr (0.10 compared to 0.08, relative increase of 125%; p = 0.04) from baseline. There were no significant changes after 30 seconds of exercise. After 60 seconds of exercise, MELAS subjects had significantly increased Pi/PCr (mean 0.74 compared to 0.47, relative increase of 157%; p = 0.01). After 5 minutes of exercise, torque was significantly increased (3839 compared to 1426 N*M, relative increase of 269%; p = 0.03) (Torque in (N*M) = Power (watts)/[2π (rad/rev) X ω (rev/s) X 60 s/min]. There were no other significant changes at 5 minutes.

**Table 4:** Effect of L-arginine on muscle metabolism during rest and different exercise regimens in MELAS subjects as measured by ^31^P-MRS (mean ± SD).

Rest	Baseline (no L-arginine) Mean ± SD	Single dose L-arginine Mean ± SD	P (t-test): change from baseline following single dose L-arginine	Steady state L-arginine Mean ± SD	P (t-test): change from baseline on 6 wk steady state L-arginine
PCr	30.6 ± 1.8	29.2 ± 0.8	0.30	28.2 ± 1.6	0.20
Pi	2.44 ± 0.40	2.78 ± 0.56	0.10	2.89 ± 0.30	0.06
Sum ATP	6.52 ± 0.81	6.60 ± 0.16	0.89	7.96 ± 1.69	0.38
pH	6.87 ± 0.06	6.89 ± 0.02	0.63	6.89 ± 0.03	0.54
pMg	2.87 ± 0.10	2.87 ± 0.05	0.94	2.87 ± 0.14	0.96
[Mg ^2+^]	1.45 ± 0.28	1.41 ± 0.14	0.88	1.41 ± 0.36	0.92
Pi/PCr (ADP)	0.08 ± 0.02	**0.10 ± 0.02**	**0.04** *	0.10 ± 0.01	0.12
**30 s**					
PCrRec ½ (s)	38.3 ± 12.0	25.6 ± 1.6	0.23	32.6 ± 11.7	0.60
Pi (B)	6.43 ± 2.76	8.05 ± 0.33	0.44	**11.8 ± 1.47**	**0.02** *
Pi/PCr (ADP)	0.31 ± 0.20	0.42 ± 0.05	0.37	**0.76 ± 0.20**	**0.008** *
HEP power	0.23 ± 0.18	0.32 ± 0.08	0.33	0.41 ± 0.04	0.15
HEP dpH	0.16 ± 0.11	0.22 ± 0.14	0.14	0.22 ± 0.05	0.49
Watts/RPM	0/11.5	0/11.3		0/9.3	
**60s**					
AnPwr	0.14 ± 0.03	0.16 ± 0.04	0.30	0.16 ± 0.02	0.18
dpH	0.55 ± 0.33	0.58 ± 0.39	0.48	0.43 ± 0.18	0.37
Pi (B)	7.78 ± 2.61	10.13 ± 1.52	0.13	11.48 ± 0.98	0.18
Pi/PCr (ADP)	0.47 ± 0.27	**0.74 ± 0.29**	**0.01** *	0.85 ± 0.15	0.19
PCr rec ½ (s)	29.3 ± 11.0	56.1 ± 54.2	0.42	38.1 ± 6.47	0.26
Watts/RPM	8.2/13	8.3/9.3		6.7/7	
**5 min**					
ATP (Prod)CK	0.16 ± 0.06	0.16 ± 0.03	0.86	0.17 ± 0.03	0.62
ATP (Prod)Ox	0.31 ± 0.11	0.33 ± 0.07	0.78	0.34 ± 0.05	0.61
ATP(Prod)An	0.75 ± 0.42	0.80 ± 0.44	0.78	0.74 ± 0.16	0.95
ATP(Prod)tot	1.22 ± 0.60	1.30 ± 0.55	0.78	1.25 ± 0.22	0.89
dpH	0.32 ± 0.21	0.34 ± 0.26	0.86	0.28 ± 0.10	0.67
Pi/PCr (ADP)	0.95 ± 0.86	0.99 ± 0.59	0.88	1. 14 ± 0.41	0.74
PCrRec ½ (s)	64.4 ± 35.0	63.3 ± 34.7	0.80	41.7 ± 1.57	0.39
Watts/RPM	4.2/11.2	6.7/9.7		5/9.3	

Key: AnPwr = anaerobic power; ATP = adenosine triphosphate; ATPAn = ATP anaerobic; ATPOx = ATP oxidative

ATPtot = ATP total; HEP = high energy phosphates; [Mg2+] = intracellular magnesium

pMg = free or paracellular magnesium and is calculated by the formula—log_10_[Mg2+]

Pi = inorganic phosphate; Pi/PCr ratio = ADP ratio; PCr = phosphocreatine

PCrRec 1/2 = halftime of phosphocreatine recovery in seconds; RPM = rotations per minute; SD = standard deviation

P is two-tailed P-value from paired t-test

* statistically significant result

#### Steady state L-arginine

At rest, there were no significant changes in any of the parameters from baseline. Following 30 seconds of exercise, MELAS subjects had significantly increased inorganic phosphate (Pi) (11.8 compared to 6.43, relative increase of 183%; p = 0.02) and Pi/PCr (ADP) (0.76 compared to 0.31, relative increase of 245%; p = 0.008) when compared to baseline. There were no significant changes after 60 seconds or 5 minutes of exercise. There also appeared to be a trend toward a blunted decrease in pH (less acidosis) after 60 seconds and 5 minutes of exercise which did not reach statistical significance.

**Fig 4:**
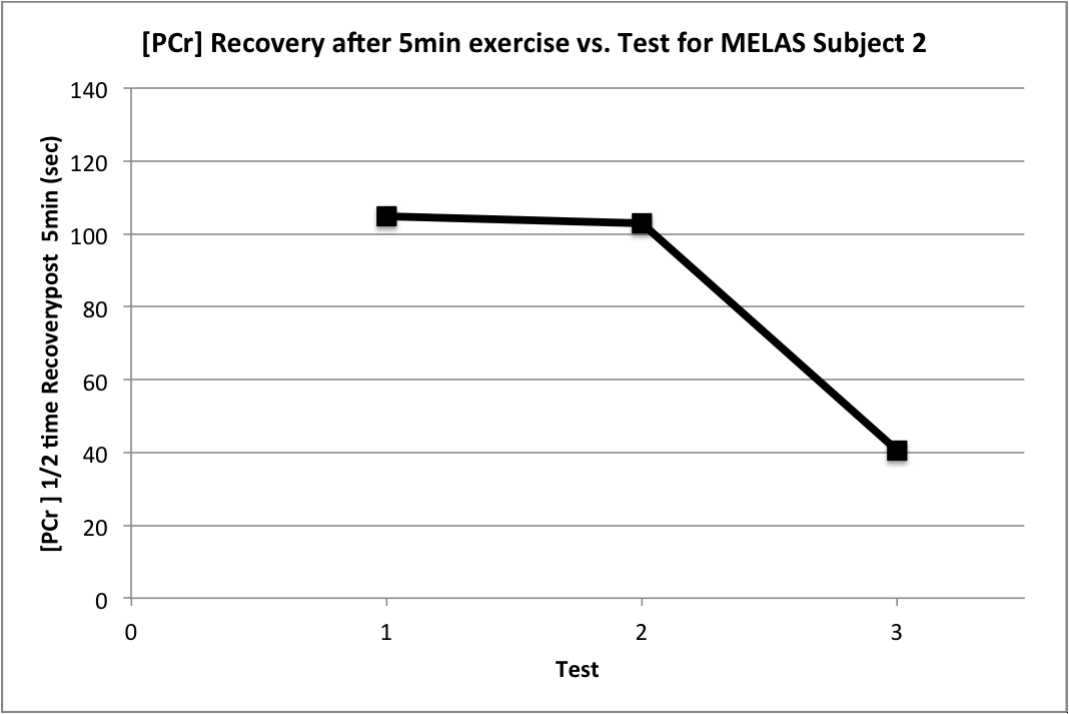
Half-time of phosphocreatine (PCr) recovery (in seconds) following 5 minutes of moderate-intensity aerobic exercise protocol in MELAS subject 2 at baseline (test 1- no L-arginine) and following single-dose L-arginine (test 2) and 6 week steady-state L-arginine (test 3) therapy.

The response of MELAS subject 2 to L-arginine supplementation deserves special attention. This subject showed an extraordinary reduction in half time of phosphocreatine recovery following 5 minutes of exercise on maintenance dosing of arginine (**Fig 4**), with a reduction from 104.8 to 40.5 mM/min (mean in controls was 35.3 mM/min). This effect was not observed following 30 and 60 seconds of exercise, nor was it observed following single dosing of L-arginine.

## Discussion

We found that subjects with MELAS had lower ATP levels, increased phosphocreatine (PCr) levels, and elevated PCr/ATP ratios at rest compared to controls (which were statistically significant on unpaired t-test analysis but not on nonparametric Kruskal-Wallis analysis). This is in contrast to decreased PCr levels and PCr/ATP ratios reported in the literature in mitochondrial myopathies; however, the latter studies were not restricted to MELAS syndrome [24]. These results suggest a favorable energetic state of muscle in our patients at rest compared to our study controls of uncertain etiology.

Cytosolic free concentration of magnesium is calculated from ^31^P MRS using the chemical shift of ß-ATP. We found that intracellular magnesium concentrations in muscle were lower in MELAS subjects compared to controls with high significance. Decreased intracellular free magnesium in *brain* has been previously reported in mitochondrial cytopathy and pediatric migraine [25]; however, to our knowledge, decreased intracellular magnesium in *muscle* has never been reported in mitochondrial disease. Magnesium is a cofactor for a number of enzymes involved in energy transduction, and influences the amount of energy released through ATP hydrolysis as MgATP^2-^ [26]. In addition, magnesium is vital for normal muscle contraction/relaxation by competing with calcium for binding to troponin, parvalbumin, and myosin. Magnesium requires active, energy dependent transport into myocytes [26]. Previous authors have hypothesized that intracellular magnesium is decreased in mitochondrial cyopathy to re-equilibrate free energy produced through ATP hydrolysis in the face of increased Pi and ADP [25]; however, our MELAS subjects at rest had reduced Pi and Pi/PCr compared to controls, making this an unlikely explanation in our cohort. Another potential explanation for the low intracellular magnesium levels we observed is insufficient energy for intracellular magnesium transport. It is possible that intracellular hypomagnesemia may be a contributing factor to the myopathy in MELAS. In addition, this raises the question of whether individuals with MELAS also have intracellular hypomagnesemia in other tissues, including neurons, endothelial cells, and vascular smooth muscle and whether this may play a role in stroke-like episodes. Given the role of magnesium in vasodilation and mitochondrial membrane stability, and the association of hypomagnesemia with cerebrovascular accident and migraine, this is an important consideration for future study [27]. Recently, magnesium sulfate in association with the ketogenic diet was used successfully to decrease the frequency of stroke-like episodes in a patient with MELAS [28].

In the first 30 seconds of exercise, energy is produced primarily through high energy phosphates via creatine phosphokinase. After 1 minute of high intensity exercise, anaerobic glycolysis plays an increasing role, and after 5 minutes of moderate intensity exercise, energy is derived predominantly from oxidative phosphorylation through the mitochondrial respiratory chain [7]. We found that following 30 s and 60 s of exercise, the Pi/PCr ratio, corresponding to levels of intracellular ADP, was decreased in MELAS subjects compared to controls. We hypothesize that lower levels of ADP (i.e. relatively higher levels of non-hydrolyzed ATP) represent a lower capacity for work in the MELAS subjects, and this is corroborated by decreased measured torque. Arginine supplementation (single dose and maintenance) significantly increased Pi/PCr at 30 and 60 seconds, and increased torque (not statistically significant).

Post-exercise phosphocreatine recovery is reported as the most sensitive abnormality on ^31^P-MRS of muscle in mitochondrial myopathy, since it is purportedly entirely achieved through oxidative phosphorylation [6]. We did not find any significant differences in PCr recovery between MELAS subjects and controls as a group following 30s, and 60s of exercise. However, there was a statistically significant delay in PCr recovery following 5 minutes of exercise in the MELAS subjects compared to healthy controls using nonparametric analysis (Kruskal-Wallis test) which was not supported by unpaired t-test analysis. This may be best interpreted as a trend and may be a reflection of the limitation of the small sample size of this pilot study. Individually we found that MELAS subject 2 had an extremely delayed PCr recovery at 5 minutes which was approximately twice as long as that of controls. This subject had a remarkable normalization of PCr recovery following maintenance dosing of L-arginine (**Fig 4**). Although this subject had an intermediate mutation burden in blood (41%), she appeared to be the most affected in *muscle* based on her results on graded cycle ergometry and ^31^P-MRS studies. It is possible that the benefits of arginine are greater in subjects with more severe muscle disease. Given the variable tissue-specific heteroplasmy of mutant to wild-type mitochondrial DNA in mtDNA inherited disorders, it is highly probable that MELAS subject 2 had a higher % of mutant mtDNA in muscle than her siblings. That being said, we cannot exclude that the latter changes on ^31^P-MRS were due to chance fluctuations in the patient’s underlying myopathy.

At increasing speeds or intensity levels of exercise, the anaerobic threshold is the point above which the muscles derive the majority of their energy more from anaerobic glycolysis than aerobic oxidation and there is an accumulation of blood lactate and hydrogen ions from pyruvate which is produced faster than it can be used aerobically, leading to metabolic acidosis, fatigue and muscle pain. The anaerobic threshold may also be considered as the highest intensity of exercise at which aerobic oxidation is the primary energy source. Aerobic metabolism is dependent upon a combination of diffusion of O_2_ in the lungs, the O_2_ carrying capacity of the blood (Hb), cardiovascular delivery of O_2_ to the muscle (perfusion), and O_2_ extraction by the muscle. In our MELAS subjects, pulmonary function/oxygen saturations, cardiac function and Hb were within normal limits.

On graded cycle ergometry, we demonstrated a statistically significant lower mean percentage of VO2_peak_ reached during exercise in our MELAS siblings compared to matched healthy controls as predicted. There was a trend toward increased ventilation relative to oxygen uptake in all three MELAS subjects, although this did not reach statistical significance (VE/VO2 of 35 ± 7.0 versus 27 ± 2.1 in controls; p = 0.083).

As previously mentioned, MELAS subject 2 appeared most affected in muscle. This subject had the most exaggerated increase in ventilation relative to oxygen extraction. She also demonstrated the greatest reductions in VO_2_ (15.3 ml/kg/min; controls 34.2 ± 9.7), peak work capacity (1.52 watts/kg; controls 2.64 ± 0.81), and percentage of maximum work at anaerobic threshold (29%; controls 65.2 ± 14.7%).

In a prior study of 40 patients with mitochondrial myopathy using maximal cycle exercise, patients demonstrated significantly lower mean peak work capacity, oxygen uptake (VO_2_) and mean peak systemic arteriovenous oxygen difference [8]. Their increase in cardiac output relative to VO_2_ and ventilation were exaggerated and there was an inverse relationship between proportion of skeletal muscle mutant mtDNA and peak 0_2_ extraction during exercise.

L-arginine supplementation significantly increased the percentage of maximum work performed at anaerobic threshold in the MELAS sibs. In addition, the administration of 6 weeks of L-arginine therapy resulted in an increase in the mean percentage of maximum heart rate at the anaerobic threshold in the MELAS sibs (82.0 ± 13.0 versus baseline of 76.7 ± 13.3; p = 0.067), which approached statistical significance. When the results from single and maintenance dosing of L-arginine were averaged, the percentage of maximum work at the anaerobic threshold was significantly increased from baseline in the MELAS siblings (51% compared to 40.7%; p = 0.037). These results in the aggregate suggest an improvement in aerobic metabolism in the MELAS subjects in response to L-arginine that was apparent despite the small sample size.

Arginine is a dibasic, semi-essential amino acid. Arginine is a precursor for a number of important biochemical reactions. It is converted to nitric oxide through the action of endothelial nitric oxide synthase, which plays an important role in vasodilation [29]. Arginine is also a precursor for creatine biosynthesis. In addition, arginine can be converted to agmatine, which serves as a precursor in the polyamine pathway, acts as a neurotransmitter, and inhibits nitric oxide synthase [29]. Finally, arginine can be converted to the tricarboxylic acid cycle intermediate alpha-ketoglutarate, enhancing cycle kinetics (anapleurosis) [29]. We have demonstrated that patients with MELAS have a relative hypoarginemia, and this is supported by the literature [3]. Arginine supplementation may address this deficiency; however, it remains unclear through which of these mechanisms arginine improves muscle metabolism in patients with MELAS. It has been previously suggested that L-arginine supplementation in MELAS may work by enhancing nitric oxide mediated vasodilation of blood vessels with intrinsically impaired capacity for vasodilation [4]. A recent study found elevated baseline levels of intracellular nitric oxide in m.3243A>G cybrid cells, bringing into dispute the latter role of L-arginine supplementation and raising the possibility of an alternative metabolic role [30]. Our finding of improved aerobic metabolism supports a benefit from arginine supplementation beyond nitric oxide mediated vasodilation. We did not find that intracellular phosphocreatine concentration was increased with arginine supplementation, making increased creatine synthesis a less likely explanation. Increased anaplerosis by conversion of arginine into glutamate, and ultimately the Kreb cycle intermediate alpha-ketoglutarate is an attractive possibility. L-arginine supplementation has been used successfully to treat mitochondrial cardiomyopathy by enhancing citric acid cycle kinetics independent of myocardial blood flow [31].

A significant limitation to our study was our small sample size, which had insufficient power to allow us to detect small differences between MELAS subjects and controls. We also did not measure changes in muscle perfusion with L-arginine supplementation.

In conclusion, it appears that L-arginine supplementation produces some benefit in total body aerobic capacity and muscle metabolism in MELAS syndrome based on *in vivo* laboratory exercise testing. The mechanisms underlying these improvements are not yet elucidated and warrant further study. It is not clear at this time whether these improvements translate into “real world” functional benefits. Furthermore, our results give some insight into a possible non-vascular role of L-arginine in the treatment and prevention of stroke-like episodes in MELAS syndrome. Finally, the significant intracellular hypomagnesemia in muscle is a novel finding warranting further exploration, and raises the question of the potential efficacy of magnesium supplementation in MELAS syndrome.

## Supporting Information

S1 Protocol (PDF)

S1 TREND Checklist (PDF)

## References

[1] Sproule DM, Kaufmann P. Mitochondrial encephalopathy, lactic acidosis, and stroke-like episodes: basic concepts, clinical phenotype, and therapeutic management of MELAS syndrome. Ann N Y Acad Sci. 2008; 1142: 133–158. 10.1196/annals.1444.011 18990125

[2] Koga Y, Povalko N, Nishioka J, Katayama K, Yatsuga S, Matsuishi T. Molecular pathology of MELAS and L-arginine effects. Biochim Biophys Acta. 2012; 1820: 608–614. 10.1016/j.bbagen.2011.09.005 21944974

[3] Koga Y, Povalko N, Nishioka J, Katayama K, Kakimoto N, Matsuishi T. MELAS and L-arginine therapy: pathophysiology of stroke-like episodes. Ann N Y Acad Sci.2010; 1201: 104–110. 10.1111/j.1749-6632.2010.05624.x 20649546

[4] Koga Y, Akita Y, Junko N, Yatsuga S, Povalko N, Fukiyama R, et al Endothelial dysfunction in MELAS syndrome improved by L-arginine supplementation. Neurology. 2006; 66: 1766–1769. 16769961 10.1212/01.wnl.0000220197.36849.1e

[5] Argov Z, Bank WJ. Phosphorus magnetic resonance spectroscopy (31P MRS) in neuromuscular disorders. Ann Neurol. 1991; 30:90–97. 1834009 10.1002/ana.410300116

[6] Mattei JP, Bendahan D, Cozzone P. P-31 magnetic resonance spectroscopy. A tool for diagnostic purposes and pathophysiological insights in muscle diseases. Reumatismo. 2004; 56:9–14. 15105904 10.4081/reumatismo.2004.9

[7] Volpi L, Ricci G, Orsucci D, Alessi R, Bertolucci F, Piazza S, et al Metabolic myopathies: functional evaluation by different exercise testing approaches. Musculoskelet Surg. 2011; 95:59–67. 10.1007/s12306-011-0096-9 21373907

[8] Taivassalo T, Jensen TD, Kennaway N, DiMauro S, Vissing J, Haller RG. The spectrum of exercise tolerance in mitochondrial myopathies: a study of 40 patients. Brain. 2003;126:413–23. 12538407 10.1093/brain/awg028

[9] Geary GG, Krause DN, Duckles SP. Estrogen reduces myogenic tone through a nitric oxide-dependent mechanism in rat cerebral arteries. Am J Physiol. 1998; 275:H292–300. 9688926 10.1152/ajpheart.1998.275.1.H292

[10] Wells GD, Heale L, Schneiderman JE, Wilkes DL, Atenafu E, Coates AL, et al Assessment of body composition in pediatric patients with cystic fibrosis. Pediatr Pulmonol. 2008; 43:1025–1032. 10.1002/ppul.20913 18781652

[11] Miller MR, Hankinson J, Brusasco V, Burgos F, Casaburi R, Coates A, et al Standardisation of spirometry. Eur Respir J. 2005; 26:319–338. 16055882 10.1183/09031936.05.00034805

[12] Corey M, Levison H, Crozier D. Five- to seven-year course of pulmonary function in cystic fibrosis. Am Rev Respir Dis. 1976; 114:1085–1092. 1008344 10.1164/arrd.1976.114.6.1085

[13] Godfrey S, Davies CT, Wozniak E, Barnes CA. Cardio-respiratory response to exercise in normal children. Clin Sci. 1971; 40:419–431. 5556096 10.1042/cs0400419

[14] Hay JA, Cairney J. Development of the habitual activity estimation scale for clinical research: a systematic approach. Pediatr Exerc Sci. 2006; 18:193–202

[15] Wells GD, Wilkes DL, Schneiderman-Walker J, Elmi M, Tullis E, Lands LC, et al Reliability and validity of the habitual activity estimation scale (HAES) in patients with cystic fibrosis. Pediatr Pulmonol. 2008; 43:345–353. 10.1002/ppul.20737 18306334

[16] Jones NL. Clinical exercise testing WB Saunders, Philadelphia 4th edition 1997; pp 243

[17] Banks L, Wells GD, McCrindle BW. Cardiac energy metabolism is positively associated with skeletal muscle energy metabolism in physically active adolescents and young adults. Appl Physiol Nutr Metab. 2014; 39: 363–368. 10.1139/apnm-2013-0312 24552379

[18] Wells GD, Wilkes DL, Schneiderman JE, Rayner T, Elmi M, Selvadurai H, et al Skeletal muscle metabolism in cystic fibrosis and primary ciliary dyskinesia. Pediatr Res. 2011; 69:40–45. 10.1203/PDR.0b013e3181fff35f 20938370

[19] Zanconato S, Buchthal S, Barstow TJ, Cooper DM. 31P-magnetic resonance spectroscopy of leg muscle metabolism during exercise in children and adults. J Appl Physiol. 1993; 74:2214–2218. 8335550 10.1152/jappl.1993.74.5.2214

[20] Boska M. ATP production rates as a function of force level in the human gastrocnemius/soleus using 31P MRS. Magn Reson Med. 1994; 32:1–10. 8084222 10.1002/mrm.1910320102

[21] Petroff OA, Prichard JW, Behar KL, Alger JR, den Hollander JA, Shulman RG. Cerebral intracellular pH by 31P nuclear magnetic resonance spectroscopy. Neurology. 1985; 35:781–788. 4000479 10.1212/wnl.35.6.781

[22] Iotti S, Frassineti C, Alderighi L, Sabatini A, Vacca A, Barbiroli B. In vivo (31)P-MRS assessment of cytosolic [Mg(2+)] in the human skeletal muscle in different metabolic conditions. Magn Reson Imaging. 2000; 18:607–614. 10913722 10.1016/s0730-725x(00)00132-6

[23] Inbar O, Bar-Or O. Anaerobic characteristics in male children and adolescents. Med Sci Sports Exerc. 1986; 18:264–269. 3523102 10.1249/00005768-198606000-00002

[24] Taylor DJ, Kemp GJ, Radda GK. Bioenergetics of skeletal muscle in mitochondrial myopathy. J Neurol Sci. 1994; 127:198–206. 7707079 10.1016/0022-510x(94)90073-6

[25] Barbiroli B, Iotti S, Cortelli P, Martinelli P, Lodi R, Carelli V, et al Low brain intracellular free magnesium in mitochondrial cytopathies. J Cerebral Blood Flow Metab. 1999; 19:528–532. 10326720 10.1097/00004647-199905000-00007

[26] Flatman PW. Mechanisms of magnesium transport. Annu Rev Phvsiol. 1991; 53:259–71. 2042962 10.1146/annurev.ph.53.030191.001355

[27] Volpi SL. Magnesium in disease prevention and overall health. Adv Nutr. 2013; 4:378S–383S. 10.3945/an.112.003483 23674807 PMC3650510

[28] Steriade C, Andrade DM, Faghfoury H, Tarnopolsky MA, Tai P. Mitochondrial encephalopathy with lactic acidosis and stroke-like episodes (MELAS) may respond to adjunctive ketogenic diet. Pediatric Neurology. 2014; 50:498–502. 10.1016/j.pediatrneurol.2014.01.009 24656211

[29] Morris SM Jr. Arginine metabolism: boundaries of our knowledge. J Nutr. 2007; 137: 1602S–1609S. 17513435 10.1093/jn/137.6.1602S

[30] Gamba J, Gamba LT, Rodrigues GS, Kiyomoto BH, Moraes CT, Tengan CH. Nitric Oxide Synthesis Is Increased in Cybrid Cells with m.3243A>G Mutation. Int J Mol Sci. 2012;14:394–410. 10.3390/ijms14010394 23263669 PMC3565270

[31] Arakawa K, Kudo T, Ikawa M, Morikawa N, Kawai Y, Sahashi K, et al Abnormal myocardial energy-production state in mitochondrial cardiomyopathy and acute response to L-arginine infusion. C-11 acetate kinetics revealed by positron emission tomography. Circ J. 2010; 74:2702–2011. 21048330 10.1253/circj.cj-10-0044

