# Supplementary material for: L-Arginine Affects Aerobic Capacity and Muscle Metabolism in MELAS (Mitochondrial Encephalomyopathy, Lactic Acidosis and Stroke-Like Episodes) Syndrome
Source: PLoS One. 2015 May 20;10(5):e0127066. doi: 10.1371/journal.pone.0127066 (PMC4439047; doi:10.1371/journal.pone.0127066)
Supplement: S1 Protocol — (PDF) [file pone.0127066.s001.pdf]

Pilot study to investigate the efficacy of L-arginine therapy on endoethelium-dependent vasodilation & mitochondrial metabolism in MELAS syndrome.

STUDY PROTOCOL

Version Date: October 14, 2011

Principal Investigator: Ingrid Tein, MD
Director Neurometabolic Clinic & Research Lab
Division of Neurology, Hospital for Sick Children

Co-Investigators: Dr. Greg Wells
Dr. Lance Rodan
Dr. Ian Wilson

PROJECT SUMMARY

MELAS patients suffer from exercise intolerance, weakness, poor vision or blindness, poor growth, developmental delay, and deafness. They also have unique 'stroke-like' episodes (SLEs) which are not due to blockages of large or medium arteries. These 'strokes' are thought to be due to energy failure of very small brain blood vessels combined with energy failure in the mitochondria (cell battery) of the brain cells, especially in the back region of the brain in the vision centre. This leads to visual loss and paralysis. The overall goal of this grant is to better understand the mechanism of these SLEs at the level of the brain cells and small blood vessels. To do this, we will study a family of 4 siblings, each with different severities of MELAS, using safe, non-invasive tests. We will determine whether there is a decrease in the ability of small brain blood vessels to increase blood flow by dilating in response to certain strong stimuli such as increased blood carbon dioxide levels or in response to brain cell activation in the vision centre by strong visual stimuli. We will use a technique called BOLD-fMRI which can detect changes in brain blood flow. As exercising muscle also depends on increased blood flow and mitochondrial energy, we will study different measures of aerobic energy metabolism in exercising muscle using cycle exercise testing and special phosphorus-magnetic resonance spectroscopy which measures the changes in the major chemicals of muscle energy metabolism. The dietary amino acid L-arginine is known to dilate blood vessels increasing blood flow and to decrease toxic free radicals that are generated by dysfunctional mitochondria. We will determine the effect of a single dose and a 6 week trial of L-arginine, on brain blood vessel reactivity, brain cell activation and muscle aerobic function to see how useful this would be in the treatment of these patients and other mitochondrial disorders which present with strokes.

RATIONALE

Nitric oxide (NO) is synthesized from the amino acid L-arginine by nitric oxide synthases (NOS). **A.** Tonic release of NO is a significant regulator of cerebral blood flow and is responsible for vasodilation (1). In brain, the relative importance of neuronal nNOS in neurons or perivascular nerves vs endothelial eNOS in cerebral vessels in the control of cerebral blood flow is unknown (1). NO generated by eNOS diffuses from the endothelium to smooth muscle where it increases cGMP leading to smooth muscle relaxation.**B**.NO also protects against peroxide-mediated cytotoxicity from reactive oxygen species (ROS) (2). Because MELAS (Mitochondrial Encephalomyopathy, Lactic Acidosis, Stroke-like episodes) patients have decreased L-arginine levels (3,4), vasodilatation may be significantly decreased. **C.** Further, as MELAS individuals have defective respiratory chain activity, a high NADH/NAD ratio could inhibit NOS further decreasing NO production. **D**. In addition, defective respiratory chain function in enlarged mitochondria documented in cerebrovascular endothelial and smooth muscle cells in MELAS (5,6) with bioenergetic compromise could also lead to aberrant vascular tone/autoregulation, resulting in local ischemia (7). **E**. Also, most MELAS mutations result in deficient Complex I activity (8) which may be associated with increased ROS production (9). Studies of acute and chronic L-arginine therapy in MELAS have suggested significant alleviation and potential prevention of stroke-like episodes (SLEs) (3,10,11).Improved cerebral hypoperfusion by SPECT scanning & major clinical improvement was demonstrated in one patient at the third SLE 2 hrs after intravenous (iv) L-arginine and in another patient on chronic L-arginine (10). Flow mediated vasodilation of brachial artery diameter as measured by ultrasound also showed improvement in MELAS patients 2 hrs after iv L-arginine (10). Thus, L-arginine may reduce ischemic damage in acute focal brain ischemia by (a) increasing microcirculation (12), (b) decreasing ROS damage (c) modulating neurotransmitters (13). In muscle, it may also increase capillary perfusion improving exercise capacity (14). Understanding the mechanisms of action of L-arginine in MELAS will foster development of more targeted therapies for this and other mitochondrial defects.

SIGNIFICANCE

The current study has the potential to significantly improve our understanding of the molecular and vascular pathophysiology of MELAS which is not well understood at present. Recent work suggests neuronal cytopathy, small vessel angiopathy or a combination of the two are responsible for SLEs (15). If we can identify the fundamental underlying defect(s) in cerebrovascular, neuronal and muscle oxidative function in MELAS, we can recommend more targeted therapies to improve health, quality of life, and survival in these individuals. We will also have identified a tool that may help predict the risk of SLEs. This study will allow us to

quantify the effect of L-arginine in MELAS patients and to correlate the physiological responses of muscle as measured by ^{31}P MRS and the cerebrovascular response as measured by BOLD-fMRI. This will also have applications to individuals with secondary mitochondrial failure.

STUDY AIMS

1. To determine the efficacy and primary mechanism (s) of action of L-arginine in MELAS syndrome by quantifying the effect of L-arginine on (a) cerebrovascular reactivity (CVR) to CO_2 stimulus (b) neuronal cell activation of CVR and (c) muscle oxidative function in MELAS siblings with the common 3243 A>G mutation.
2. To determine the relative contributions of impaired microvascular endothelial cell function vs impaired neuronal cell activation of CVR in MELAS syndrome.
3. (a) To determine the efficacy of BOLD-fMRI (tool to measure CVR) to differentiate between impaired CVR in a family with variable CNS severity of MELAS compared to controls (b) to determine whether a relationship exists between the severity of neurological phenotype and degree of impairment of CVR as a prognostic predictor for risk of stroke. (* % mutant mtDNA blood may not correlate with % mutant mtDNA in brain, muscle or heart).

STUDY PLAN

Brain BOLD-fMRI allows us to examine CVR non-invasively *in vivo* in 4 MELAS sibs vs 4 age-, sex- and menstrual cycle- matched normal controls. To determine the relative contributions of (a) impaired isolated microvascular endothelial cell function in response to changes in PCO_2 (using a rebreathing circuit) vs (b) impaired occipital neuronal cell (in response to a strong visual stimulus) activation of CVR, we will study both paradigms using brain BOLD-fMRI. Given the variable severity of neurological phenotypes in this family, we will determine the relationship between the severity of the phenotype and the degree of dysfunction in CVR to PCO_2 vs neuronal stimulus. This correlation may provide a useful tool for predicting the risks for SLEs. To determine the correlation between brain and muscle dysfunction, we will compare brain BOLD-fMRI findings with parameters of total body and muscle oxidative function as determined by maximal incremental cycle ergometry and ^{31}P-MRS of exercising muscle, respectively. Following these baseline studies, we will then quantify the effect, in only our MELAS sibs, of L-arginine (single oral dose & 6 wk steady state trial) on CVR, neuronal activation, and total body and muscle oxidative function to determine which function(s) is improved. Absorption and exhaled NO production and urine NOx will be measured.

PREDICTED OUTCOMES

1. We predict the severity of reduction in CVR in MELAS patients will be directly proportional to the severity of the neurological phenotype. The BOLD signal in brain will be decreased because of decreased cerebrovascular reactivity due to (a) endothelial cell dysfunction and/or (b) decreased neuronal cell activation of blood flow.
2. There will be a direct correlation between the degree of oxidative dysfunction in muscle, decrease in total body maximal aerobic capacity and impairment of CVR in MELAS which will be directly proportional to the severity of the neurological phenotype.
3. L-arginine will improve CVR to (a) CO_2 stimulus and (b) neuronal cell activation, as well as total body maximal aerobic capacity and muscle oxidative function by increasing microvascular (arteriolar) endothelium-dependent vasodilation and thus flow. L-arginine will also improve neuronal cell mitochondrial function.

HOW DATA WILL BE UTILIZED:

1. To develop parameters for an expanded clinical trial with a larger cohort of MELAS patients based upon the predicted numbers needed to establish statistical significance for the observed outcome measures. This would be based upon the trends observed in our pilot study.
2. To determine the utility and sensitivity of BOLD-fMRI (as a tool to measure CVR) to differentiate between the different severities of neurological phenotypes in a small cohort of heteroplasmic siblings and to identify trends or a threshold for the prognostic prediction for risk of stroke. This could be important also in a given individual as the % mutant mtDNA increases with aging and exogenous mitochondrial stressors (e.g. XRT, toxins, viruses).
3. To increase understanding about the relative contributions of impaired microvascular reactivity versus impaired neuronal cell activation in the pathogenesis of SLEs in MELAS syndrome to develop more targeted therapies.

STUDY DRUG:

L-arginine oral administration: L-arginine (L-arg) is approved for clinical use in MELAS by the Ontario Ministry of Health Inherited Metabolic Diseases Program. A single oral dose of 200 mg/kg of L-arg in normal controls results in a 2.7-fold increase in plasma L-arg at 1 hr, 3-fold increase at 2 hr and sustained 2.4 fold increase at 3 hr after dose, indicating good absorption & time of peak effect & safe plasma maximum of < 200 µM (16). Oral L-arg at 200 mg/kg tid for 6 weeks in a randomized double-blind placebo-controlled study resulted in a sustained increase

in exhaled NO (eNO) compared to placebo and was well tolerated. Side effects of L-arg are rare and dose-dependent; low BP, headache, asthma, chest discomfort, nausea, cramps, and diarrhea. Adverse effects to oral L-arg most commonly occur at doses > 30 g/day or 10 g/dose (17). Numerous studies with oral L-arg at 30g/day have reported no gastrointestinal effects (18). Our 4 MELAS sibs have normal lytes, glucose, renal and liver functions & no history of gastrointestinal, respiratory or cardiac problems. L-arg has been well tolerated by most up to 18 mos (3).

STUDY POPULATION:

Study Cohort and Sampling Procedures: Four siblings with MELAS (A3243G) syndrome (1 male; 3 females) aged 16-21 years, followed in the Neurometabolic Clinic at the Hospital for Sick Children (HSC) will be studied (>80 % of MELAS patients have this mutations (19)). These sibs have both the cognitive and physical capabilities of cooperating with the outlined studies. They have from 35 % to 65 % mutant mtDNA (A3243G) in blood and the relative severity of their clinical phenotypes correlate closely and directly with these percentages. The youngest sib has the highest % of mutant mtDNA and has suffered several SLEs in the occipital lobe with a partial residual hemianopsia, partial sensorineural hearing loss, selective cognitive deficits and intermittent mild lactic acidosis. The older sibs have varying degrees of hearing loss, cognitive deficits and intermittent mild lactic acidosis which also correlate directly with their % of mutant mtDNA in blood but have suffered no SLEs. The sister with lowest % mutant mtDNA has the best academic and athletic performance. Initial contact with this family has been made during regular clinic visits by their physician, Dr. Ingrid Tein,. The study has been explained to the family who have expressed strong interest in participating. The sibs will be compared to 4 age- and sex-matched controls, and female controls will be matched according to phase in menstrual cycle corresponding with their age-matched MELAS subjects. All female MELAS subjects will undergo a urine pregnancy test at the screening visit. A positive test will result in the exclusion of that subject form the study.

Controls will be asked to complete the Physical Activity Readiness Questionnaire. All studies will be IRB approved at HSC &UHN sites.

Exclusion criteria for healthy controls: migraines, metabolic disorder, medications predisposing to lactic acidosis or vasodilatation, neuromuscular/neurologic, cardiac or pulmonary disease, visual abnormalities, hypertension, anemia and prothrombotic state. Control subjects for which an MRI is contraindicated (pacemaker, ocular metal, claustrophobia, tattoos) will be excluded from the study. Controls will be screened prior to entry into study with baseline physical exam with BP & Hb (anemia could affect cerebral flow), lactate, CK, quantitative amino acids, urine

organic acids to ensure normal values. All control subjects will undergo a urine pregnancy test at the screening visit. A positive test will result in the exclusion of that subject form the study.

STUDY DESIGN:

MELAS sibs will participate in 4 test periods. Controls will be tested at baseline once only with no L-arginine (see Table 1 for Timetable of studies)

(1) **At baseline**, both untreated controls and MELAS siblings will undergo clinical and neurological exams and three sets of tests including (A) quadriceps muscle aerobic cycle ergometry in the magnet using ^{31}P-MRS with pre- and post- CKs to assess degree of muscle effort and plasma quantitative AA to determine any effect of exercise alone on AA profile, (B) maximal incremental cycle test to investigate overall maximal aerobic capacity with pre- and post- serum CKs and lactates and urine lactate/creatinine ratios to assess degree of effort, and serum quantitative AA and exhaled NO (eNO) to determine any effect of exercise alone on these parameters, and (C) BOLD-fMRI of brain to evaluate CVR with pre- and post- AA to establish a baseline. MELAS sibs and controls will have early morning urine NOx metabolites (nitrites, nitrates) measured to establish their baselines. Plasma AA are measured by our certified HSC Core Lab. As estrogen vasodilates cerebral microvessels by increasing endothelial NOS thus increasing NO (20), we will measure CVR at 0, 4 and 12 wks to coincide with the same phase of menstrual cycle in each of 3 menstruating female sibs and serum estradiol levels will be measured in each subject and their respective age-,menstrual cycle matched controls (21).

(2) **Single Oral L-Arginine Dose Trial**: Four wks after this baseline testing, the MELAS sibs will undergo a trial with a single oral dose of L-arg of 200 mg/kg and will then be retested with the three sets of tests described above. This will allow evaluation of the acute clinical response to a single dose of L-arg and to determine any impact on (a) muscle oxidative function, (b) total body maximal aerobic capacity and (c) CVR. This will also provide an opportunity to evaluate toleration of the dose and any side effects. Tests will be performed at fixed time intervals 60 mins after the oral dose of 200 mg/kg for each MELAS patient to ensure maximum serum L-arg at the time of testing, ending by 2 hrs post-dose. Plasma AA will be measured pre- and post-test procedure to determine the effect of the single oral dose on plasma arg, citrulline and ornithine concentrations as a measure of the efficacy of absorption and to correlate the performance on testing with the concomitant plasma arg concentrations. Following the maximal graded cycle test, (i) exhaled NO (eNO) will be measured to evaluate utilization of L-arg in NO production post oral dose as well as (ii) urinary NO metabolites.

(3) **6 wk Steady State L-Arginine Trial:** If the single oral dose of L-arginine is well tolerated, the MELAS sibs will then be treated with oral L-arginine at 200 mg/kg three times daily (maximum

10g three times daily) for 6 weeks (beginning 2 wks after the single oral dose trial) to assess their clinical response to steady state L-arginine therapy (22,23). The dosage and duration of administration is comparable to studies using similar formulations (3,10,11,16). Preparation of L-arginine will be of high-grade quality prepared by our HSC Institutional Pharmacy according to regulatory standards set by the Ontario Ministry of Health Inherited Metabolic Diseases Program. For the duration of treatment, patients will be evaluated weekly by their pediatrician, Dr. I. Wilson for any adverse events. A medical questionnaire will be completed and full clinical exam including BP. Adverse events will be reported immediately to the PI and External Safety Monitoring Committee composed of a Staff Neurologist with expertise in BOLD-fMRI and Staff Metabolic/Geneticist. If 200 mg/kg is not well tolerated,dosing may be reduced by 50 % to 100 mg/kg 3X daily and the individual reevaluated. After 6 weeks of oral L-arg, MELAS sibs will undergo the same 3 sets of tests to evaluate clinical responses and biochemical parameters to steady state plasma arg levels including clinical and neurological evaluations. L-arg will then be stopped.

(4) **8-wk Wash-out:** The L-arginine supplementation will be slowly stopped over 6 days time. The schedule for decreasing the dose will be ¾ dose for 2 days, ½ dose for 2 days and then ¼ dose for 2 days and then stopping the L-arginine. Following discontinuation, the sibs will be seen every 2 weeks by their pediatrician to document any adverse effects upon withdrawal of L-arg. They will be seen for clinical and neurological exams by our Team, 8 wks after withdrawal, and will have baseline biochemical tests to ensure there are no sustained adverse events.

ANALYSIS:

The following comparisons will be made (**1**) MELAS sibs will be compared to themselves both on L-arginine (single oral dose and 6 wk steady state trial) vs. off L-arginine (pre-trial baseline) to evaluate effects of treatment. (**2**) They will be seen post L-arginine therapy to determine if there are lasting effects up to 8 wks vs. off L-arginine (baseline). (**3**) MELAS sibs at baseline vs. controls (**4**) MELAS sibs on L-arginine (single oral dose and 6 wk steady state trial) vs. controls. Four different end-points will be compared (**A**) ^{31}P-MRS study of quadriceps for PCr recovery and ATP levels (**B**) maximal oxygen uptake VO_{2max} (ml O_2/kg/min) and mean peak work capacity (Watts/kg) (**C1**) CVR to C02 stimulus (**C2**) CVR to neuronal stimulus. Comparisons will be made of the different parameters and to determine whether there are clinically significant differences between the MELAS sib groups (treated vs untreated), and MELAS sib vs control groups (Student's t-test). We understand that the sample size has insufficient power to detect statistically significant differences. However estimates of means and SD's obtained from this pilot study will be used in a future study to determine adequate sample sizes to detect statistically significant differences in these parameters.

A. ^{31}P-Magnetic resonance spectroscopy protocol – Muscle function investigation: We will study exercising quadriceps using our MR-compatible up-down ergometer and our well established aerobic exercise protocol at 65 % of maximal voluntary contraction (25). Testing will be done between 60 to 105 minutes after an oral L-arg dose of 200 mg/kg to ensure maximum serum arg at time of exercise. **Predicted outcome**: Untreated sibs: ^{31}P-MRS of quadriceps will show a delay in PCr recovery/resynthesis after exercise, reflecting the reduced ATP synthesis rate compared to controls. We predict that the degree of delay in PCr recovery will be directly proportional to the % mutant mtDNA in blood. Following L-arginine, we predict a faster PCr recovery/resynthesis after exercise and increased ATP levels. ^{31}P-MRS data will be collected on our GE Twin Speed EXCITE™ III 12.0 1.5 Tesla imaging & spectroscopy system at HSC

B. Maximal Incremental cycle testing to determine total body maximal aerobic capacity: Maximal incremental cycle ergometry is conducted in our CardioRespiratory Exercise Lab at HSC by our established protocols (26). Serum CK and quantitative AA (for arginine, ornithine and citrulline) will be measured pre- and post- exercise as well as eNO in order to correlate aerobic exercise parameters with serum arg and eNO levels..Exercise testing will be performed between 60 - 75 min following an oral L-arg dose (200 mg/kg) to ensure maximal serum arg at time of exercise. **Predicted outcome**: Pre-treatment, maximal incremental cycle ergometry will demonstrate a decrease in maximal oxygen uptake VO_{2max} (ml O_2/kg/min) and mean peak work capacity (Watts/kg) which will be directly proportional to % mutant mtDNA (27). We also predict exaggerated circulatory and ventilatory responses to exercise that will increase exponentially in relation to increasing % mutant mtDNA. We anticipate that even though there will be a decrease in O2 uptake by exercising muscle due to defective oxidative phosphorylation, there may be an additional defect, in response to increased exercise demands, related to impaired peripheral vasodilatation, due to endothelial dysfunction of small muscle arterioles. We predict this will respond to L-arg. Alternatively, if aerobic capacity and peak power do not improve with L-arg, this may indicate that endothelium of muscle and heart arterioles is less affected than brain arterioles, given their significantly lower % of mitochondria compared to CNS vessels, and that peripheral vasodilatation is already maximal.

C. CerebroVascular Reactivity Studies – Functional MRI-Blood oxygen level dependent (BOLD) of brain

Measurement of Exhaled Nitric Oxide (eNO) and early morning (first am) NO metabolites:

All subjects will have their eNO levels measured before and after graded maximal incremental cycle ergometry. We will measure eNO levels to determine the effect of oral L-arginine on NO production in our sibs in order to confirm their ability to synthesize NO from arginine and to correlate the eNO concentrations with serum arginine concentrations. We will also measure NO metabolite concentrations in the first morning urine, as this is an accurate reflection of systemic

NO production. eNO will be measured using single breath on-line measurements for the assessment of lower airway NO (28,29). Urine NO metabolites will be detected spectrophotometrically by measuring the final stable equimolar degradation products, nitrite and nitrate (NOx) (30).

Table 1. Summary Timetable of Clinical Testing

Participant Group	Clinical Exam and Bloodwork	**A.** ^{31}P-MRS of Exercising Muscle	**B.** Maximum Graded Aerobic Cycle Test	**C.** BOLD -fMRI A-15'; B-15'
**Visit 1–wk 0**	**Baseline Clinical/Neuro exams**	Day 1 AM	Day 1 PM	Day 2
4 Controls baseline only	Hb, CK, lactate, estradiol, AA, carnitine T/F, urine OA	Pre/post CK, AA AM urine NOx	Pre/post CK, lactate, AA, eNO, urine L/Cr	Pre/post AA AM urine NOx
4 MELAS baseline	CBC,diff, platelets, lytes, lactate, BUN, Cr, glucose, Ca, PO4, AST, AP, PT, INR, carnitine total/free, estradiol	Pre/post CK, AA AM Urine NOx	Pre/post CK, lactate, AA, eNO urine lactate/creatinine (L/Cr)	Pre/post AA AM urine NOx
**L-arginine trial**	Timing of study post-dose	60-105 min	60-75 min	75-105 min
**Visit 2–wk 4 Single oral dose**	**Single Dose Response (60-105 min after oral load)**	Day 1 AM Urine NOx	Day 1 PM	Day 2 AM Urine NOx
4 MELAS	Clinical + neurological exam + serum estradiol	Pre/post CK, AA	Pre/post CK, lactate, AA, eNO, urine L/Cr	Pre/post AA
**START wk 6 L-arg in MELAS**	Questionnaire and clinical exam q 1 wk X 6 by pediatric.			
**Visit 3-wk 12 6 wks ON L-arg**	**Steady State Response** clinical + neurological exam	Day 1 AM Urine NOx	Day 1 PM	Day 2 AM Urine NOx
4 MELAS	CBC, diff, platelets, lytes, BUN, Cr, glucose, Ca, PO4, AST, AP, PT, INR, estradiol	Pre/post CK, AA	Pre/post CK, lactate, AA eNO, urine L/Cr	Pre/post AA
**STOP L- Arg -> Wash out**	Questionnaire + clinical exam q 2wk X 4 by pediatrician		Key: AA= serum quantitative amino acids to assess arginine, ornithine and citrulline; CK= serum creatine kinase; OA = organic acids; eNO= exhaled nitric oxide; NOx = NO metabolites; BOLD-fMRI: A= CO2 stimulus 15 min; B = visual stimulus 15 min; Blood samples are taken from a saline lock therefore there will be only ONE venipuncture per day X 2 days	
**Visit 4-wk 20 8wk washout**	**Wash-out period** clinical + neurological exam			
4 MELAS	CBC, diff, platelets, lytes, BUN, Cr, glucose,Ca, PO4,AA AST, AP, PT, INR, CK, lactate,	Final assessment AM Urine NOx		

ADVERSE EVENT REPORTING

All adverse events will be reported to the Hospital for Sick Children Research Ethics Board according to the Hospital for Sick Children's adverse event reporting requirements. All serious adverse drug reactions to the study medication will be reported to Health Canada within 15 calendar days or for death or life-threatening events, within 7 calendar days. In the latter case,

a follow-up report must be filed within 8 calendar days. Adverse reactions will be managed according to the Hospital for Sick Children's standard clinical management practices.

CONCOMITANT MEDICATION

There will be no changes to the cofactor/vitamin therapy that the subjects are receiving prior to enrolment in the study.

QUALITY & SAFETY ASSURANCE

This project will be monitored by the Hospital for Sick Children Clinical Research Continuing Review program during the data collection phase of the project. The aim of Continuing Review is to ensure that all SickKids researchers are maintaining the highest ethical, scientific and safety standards for all study participants, and are in compliance with all relevant SickKids policies, provincial and federal legislation, and international guidelines such as ICH- Good Clinical Practice. All studies are categorized according to the Continuing Review Matrix based on the type of study and the level of risk (I to IV) to research subjects and the Clinical Research Monitor will review at minimum 10% of the research subjects' records for study eligibility, informed consent, adherence to study protocol, reporting of adverse drug reactions and adverse events, and data quality including computer database security and storage of records. Findings of the Continuing Review will be presented to the Research Ethics Board and lead PI in a written report, and specific recommendations arising from the report will be implemented in a timely manner.

REFERENCES:

1. Andresen J, Shafi N, Bryan RM. J Appl Phys 2006; 100: 318-327
2. Wink, DA, Hanbauer, I, Krishna, et al. Proc Natl Acad Sci USA, 1993; 90: 9813-7.
3. Koga, Y, Akita, Y., Junko, N., et al. Neurology, 2005; 64: 710-2.
4. Naini A, Kaufmann P, Shanske S, et al..J Neurol Sci. 2005;229- 230:187- 93. Epub 2004 Dec 15.
5. Mizukami K, Sasaki M, Suzuki T,et al. Acta Neuropathol. 1992;83(4):449-52.
6. Gilchrist JM, Sikirica M, Stopa E, Shanske S. Stroke. 1996;27(8):1420-3.
7. Goto Y. Muscle Nerve. 1995;3:S107-12.
8. Ciafaloni, E., Ricci, E., Servidei, S., et al. Neurology, 1991; 41, 1663-1664.
9. Pitkanen S, Robinson BH. J Clin Invest. 1996;98(2):345- 51.
10. Koga, Y, Akita, Y., Junko, N., et al. Neurology, 2006; 66: 1766-9.
11. Koga, Y, Akita, Y, Nishioka, J., et al. Mitochondrion, 2007; (1-2): 133-9.
12. Morikawa, E., Moskowitz, MA, Huang, Z., et al. Stroke, 1994; 25: 429-35.
13. Hirata K, Akita Y, Povalko N, et al. Brain Dev. 2008; 30: 238-45
14. Ohta F., Takagi, T., Sato, H., Ignarro, LJ. PNAS USA, 2007; 104: 1407-11.
15. Nishioka, J., Akita, Y., Yatsuga, S., et al. Brain Dev. 2008; 30:100-5
16. Grasemann H, Grasemann C, Kurtz F, et al. Eur Respir J. 2005;25(1):62-8.
17. Evans RW, Fernstrom JD, Thompson J, et al. J Nutr Biochem. 2004;15(9):534-9.
18. Hladunewich MA, Derby GC, Lafayette RA, et al. Obstet Gynecol 2006;107(4):886-95.
19. Goto, Y., Nonaka, I., & Horai, S. (1990). Nature, 348, 651-653.
20. Diomedi M, Cupini LM, Rizzato B, et al. J Neurol Sci. 2001;185(1):49-53.
21. Geary GG, Krause DN, Duckles SP. Am J Physiol. 1998;275(1 Pt 2):H292-300.
22. Abe, K., Matsuo, Y., Kadekawa, J., et al . J Neurol Sci. 1999; 162: 65-8.
23. Allard, JC., Tilak, S., and Carter, AP. AJNR Am.J.Neuroradiol.,1988; 9, 1234-1238.
24. Vesely, A., Sasano, H., Volgyesi, G., et al. Magn Reson.Med., 2001; 45, 1011-1013.
25. Taivassalo, T., Reddy, H., Matthews, P. M. Neurol Clin 2000; 18(1): 15-34.
26. Schneiderman-Walker, J., Wilkes, D. L., Strug, L., et al. J.Pediatr., 2005; 147, 321-326.
27. Taivassalo T, Jensen TD, Kennaway N, et al. Brain. 2003;126(Pt 2):413-23. 2001.
28. Baraldi E, de Jongste JC; Eur Respir J. 2002;20(1):223-37.
29. American Thoracic Society. Recommendations for standardized procedures for the on-line and off-line measurement of exhaled lower respiratory nitric oxide and nasal nitric oxide in adults and children-1999. This official statement of the American Thoracic Society was adopted by the ATS Board of Directors, July 1999. Am J Respir Crit Care Med. 1999;160(6):2104-17.
30. Nims RW, Cook JC, Krishna MC, et al. Methods Enzymol. 1996;268:93-105.
